# The effect of different degrees of visible trephine-based foraminoplasty in PETD surgery on lumbar biomechanics: a finite element analysis

**DOI:** 10.3389/fbioe.2025.1595935

**Published:** 2025-05-27

**Authors:** Duohua Li, Wei Sun

**Affiliations:** ^1^ Graduate School of Xuzhou Medical University, Xuzhou, Jiangsu, China; ^2^ Department of Spine Surgery, Affiliated Hospital of Xuzhou Medical University, Xuzhou, Jiangsu, China

**Keywords:** Lumbar disc herniation, biomechanical, foraminoplasty, visible trephine, finite element

## Abstract

**Purpose:**

This study aimed to evaluate the effect of the degree of facet joint resection under the combined action of large-channel endoscopy and visualized trephines on lumbar biomechanics.

**Methods:**

The original CT data of a healthy male volunteer were selected. An L3-5 lumbar spine model, M0, was established via the three-dimensional finite element method. Different degrees of resection of the superior articular process of L4 were simulated via a visualized trephine during the operation, and six models were established (M1: tip resection; M2: resection of the ventral 1/3; M3: resection of the ventral 1/2; M4: resection of the ventral 2/3; M5: resection of the ventral 3/4; and M6: complete resection). Loads were applied to the model to simulate six motions of flexion, extension, left/right lateral bending, and left/right rotation. The stress distributions of the vertebral body, intervertebral disc and articular cartilage of the L3-4 segment and adjacent segments were observed.

**Results:**

Compared with M0, L4 vertebral stress was elevated in the M1 model, L4 vertebral stress was reduced in the M2 and M3 models, and L4 vertebral stress was significantly elevated in the M4, M5, and M6 models (*P* < 0.05). Compared with M0, the differences in the L3 vertebral body, L5 vertebral body, L3-4 disc, and L4-5disc stresses were not statistically significant (*P* > 0.05) in the M1, M2, and M3 models, whereas the stresses were significantly higher (*P* < 0.05) in the M4, M5, and M6 models. Compared with M0, the difference in L3-4 facet joints stress between the M1, M2 and M3 models was not statistically significant (*P* > 0.05), whereas the L3-4 facet joints stress between the M4, M5 and M6 models were significantly higher (*P* < 0.05), with a greater increase on the left facet joint.

**Conclusion:**

When more than half of the superior articular process of L4 is resected under large-channel endoscopy, the stress on the vertebral body, intervertebral disc and articular cartilage of the L3-4 segment increases, which may cause iatrogenic instability but has no significant effect on the stress on the vertebral body or intervertebral disc of adjacent segments.

## 1 Introduction

Lumbar disc herniation (LDH) is a common and frequently occurring disease in orthopedics. The symptoms are low back pain, radiating pain and numbness to the lower extremities, bladder or bowel dysfunction, sexual dysfunction, or referred pain that can occasionally mimic visceral pathologies (Zhang et al., 2024). Most patients can relieve symptoms through conservative treatments such as drugs, chiropractic, physiotherapy, and acupuncture (Linhardt et al., 2016). If conservative treatment is ineffective or symptoms of cauda equina nerve compression appear, surgical treatment is needed. Compared with conservative treatment, surgical treatment can quickly relieve symptoms such as leg pain (Zhang et al., 2023; Carr, 2020). At present, percutaneous endoscopic transforaminal discectomy (PETD) is one of the main surgical methods for treating lumbar disc herniation. It uses a transforaminal endoscope to remove the protruding nucleus pulposus through the intervertebral foramen, relieving compression and reducing the patient’s pain. This surgical method has the advantages of being minimally invasive, having a short operation time, resulting in less blood loss, a short hospital stay and high postoperative satisfaction (Ding et al., 2018; Gadjradj et al., 2022; Hoogland et al., 2008). Studies (Wu et al., 2023; Li et al., 2024) have shown that extensive resection and exposure of the facet joint may lead to significant asymmetric stress changes in the bilateral facet joints and range of motion (ROM) instability in the surgical and adjacent segments. Shi et al. (Deyo and Weinstein, 2001) reported that the disc stress of L4/L5 significantly increased in most directions of motion when more than 3/5 of the superior facet of S1 was formed from the ventral to the dorsal or 1/5 from the apex to the base.

Owing to factors such as facet joint hyperplasia and foraminal stenosis, unilateral foraminoplasty is often required during the operation to expand the working channel (surgical space) and better expose the protruding intervertebral disc, dural sac and nerve root. In this process, the facet joint will be damaged to varying degrees. With the innovation of surgical instruments, the application of trephines can grind part of the superior facet joint, expand the intervertebral foramen, and then place the working channel. However, it is impossible to precisely control the location and scope of facet joint resection. The emergence of visualized trephines has solved this problem. Using visualized trephines can control the degree of facet joint resection and reduce damage to the exiting nerve root and dorsal root ganglion during puncture and catheter placement.

95% of LDH occurs at L4-5 and L5-S1, L3-4disc herniations are relatively rare, accounting for less than 5% of clinical cases (Deyo and Mirza, 2016; Vialle et al., 2015; Wang and Wu, 2023). Many studies have investigated lumbar biomechanics after surgery for lumbar disc herniation at the L4-5 and L5-S1 segments, whereas relatively few studies have focusedon the L3-4 segment. However, the biomechanics of the L4-5 segment are not completely applicable to the L3-4 segment. Therefore, in this study, three-dimensional finite element analysis was used to analyze the impact of the degree of resection of the L3 facet joint under the combined action of large-channel endoscopy and visualization of the trephine via PETD on lumbar biomechanics. This study aimed to provide a theoretical basis for achieving better surgical outcomes and minimizing complications as much as possible.

## 2 Materials and methods

### 2.1 Patient CT data collection and lumbar spine modeling

One healthy adult male volunteer (35 years old, height 168 cm, weight 75 kg) was selected, excluding those with spinal diseases. Informed consent was signed and discussed and approved by the Ethics Committee of the Affiliated Hospital of Xuzhou Medical University (XYFY2024--KL634--01). A Siemens 64-slice spiral CT system (Siemens Sensation Open CT scanner, Siemens, Erlangen, Germany) provided by the imaging department of the Affiliated Hospital of Xuzhou Medical University was used to perform spiral scanning and tomographic image processing on the L3-L5 region of the selected subject. During scanning, the volunteer was in a supine position, and the scanning cross-section was maintained perpendicular to the long axis of the body as much as possible. The scanning parameters were as follows: voltage, 120 kV; layer thickness, 0.699 mm; and tube current, 200 Ma. The CT image data are exported in Digital Imaging and Communications in Medicine (DICOM) format for backup.

The patient’s lumbar spine CT data were imported into Mimics in DICOM format. The bones are segmented by setting the gray value threshold. The segmentation threshold is set to 226–1612, the region growth mode is set to “6-connectivity”, and the model is optimized through mask separation, filling and mask editing. The masks of each vertebra obtained via segmentation are sequentially subjected to three-dimensional reconstruction. The 3D reconstruction quality is selected as the “high” quality level. After the three-dimensional model is obtained, smoothing processing is performed, “iterations” is set to 5, and the “smooth factor” is set to 0.4. After the 3D model is reconstructed, it is saved in the STL format. The stored file is imported into Geomagic wrap, triangular patches with a side length of 1.0 mm are regenerated, and then problems such as “small holes and highly refracted edges” are repaired. When smoothing, the smoothing level is set to the intermediate value. Accurate surface processing is performed on each vertebra model to obtain a solid model of each vertebra. According to the literature (Ritzel et al., 1997), the thickness of cortical bone is 2–3 mm. The “offset” operation was used to offset the vertebral body model as a whole by 2.5 mm inward, and the cortical bone part was removed. The three lumbar vertebrae are processed in turn to obtain models of the vertebrae and cancellous bone parts, and the models are stored as part of the STEP files. The parts in SolidWorks are assembled to form an assembly. The intervertebral disc is generated between adjacent vertebral bodies through the “boss feature”, and endplates with a thickness of 1 mm are created on the upper and lower surfaces. The intervertebral disc, which is composed of the annulus fibrosus and nucleus pulposus, lies between the upper and lower endplates. First, the nucleus pulposus was added between the upper and lower endplates. The nucleus pulposus is modeled as an incompressible liquid-filled cavity. Then, four 1.5 mm thick concentric rings around the nucleus pulposus were added to form the annulus fibrosus. The fibers of the annulus fibrosus are composed of rod-like elements that can only bear tension. The fibers move in a scissor-like manner in the annulus fibrosus and form an average angle of 25°–40° with the intervertebral disc. The nucleus pulposus accounts for approximately 44% of the volume of the intervertebral disc. The center of the nucleus pulposus is located approximately 3.5 mm behind the center of the intervertebral disc. The articular cartilage between the upper and lower facet joints was constructed through “drawing sketches, stretching bosses and combinations.”

### 2.2 Establishment of the articular process resection model

**FIGURE 1:**
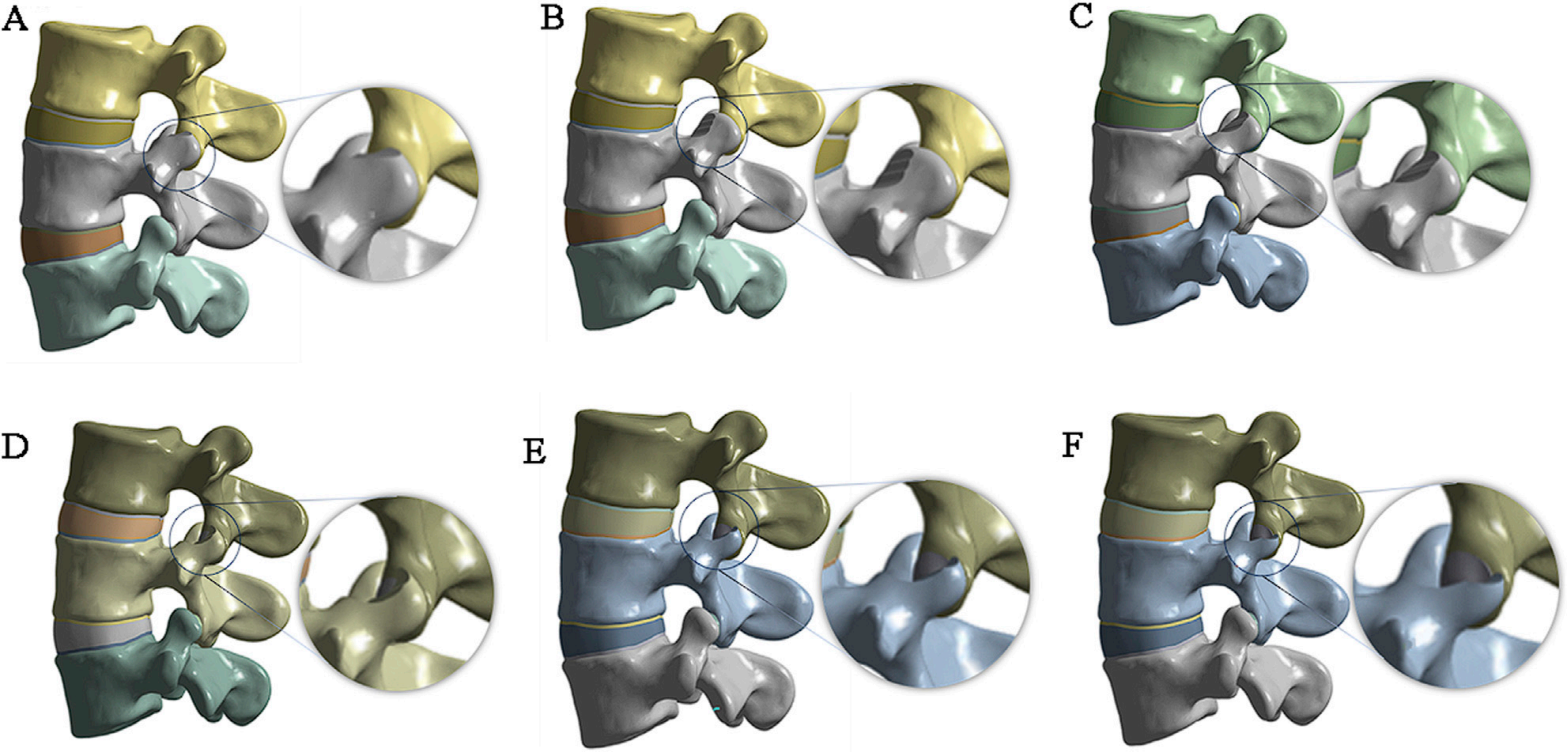
Articular process resection plan and results. **(A)** tip resection; **(B)** resection of the ventral 1/3; **(C)** resection of the ventral 1/2; **(D)** resection of the ventral 2/3; **(E)** resection of the ventral 3/4; **(F)** complete resection.

Different foraminoplasty models based on previous studies (Wu et al., 2023) and actual surgical resection of the articular process:

1 Unected model (Model 0, M0);
2 Superior facet joint apex foraminoplasty model (Model 1, M1, Figure 1A);
3 The ventral 1/3 foraminoplasty model of the superior facet joint (Model 2, M2, Figure 1B);
4 The ventral 1/2 foraminoplasty model of the superior facet joint (Model 3, M3, Figure 1C);
5 The ventral 2/3 foraminoplasty model of the superior facet joint (Model 4, M4, Figure 1D);
6 Ventral 3/4 foraminoplasty model of the superior facet joint (Model 5, M5, Figure 1E);
7 Model of complete resection of the superior facet joint (Model 6, M6; Figure 1F).

### 2.3 Building a complete model

**FIGURE 2:**
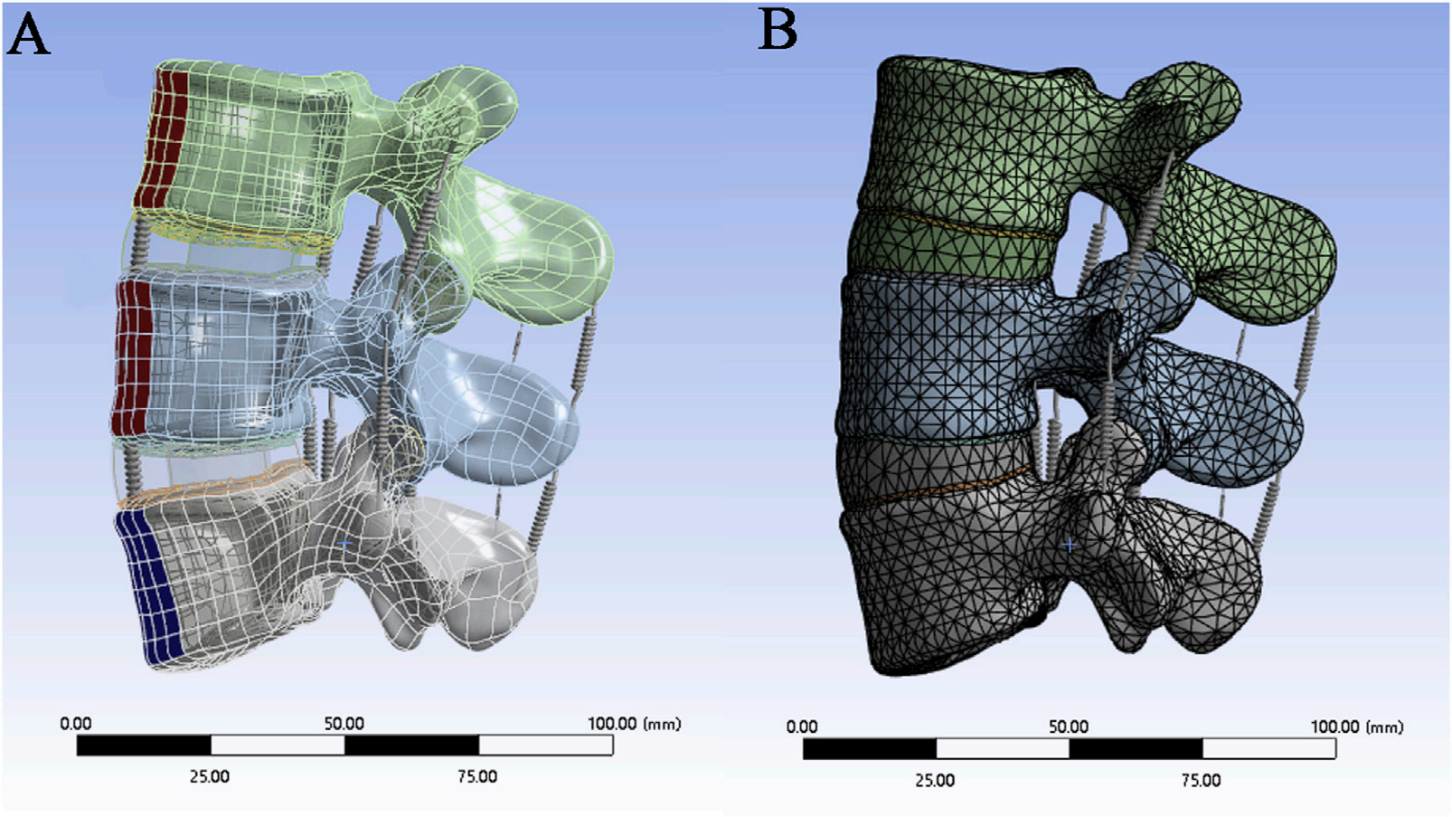
**(A)** Construction of ligaments by spring units; **(B)** Mesh division of the finite element model.

The complete three-dimensional finite element model is imported into ANSYS 21.0 for finite element analysis. spring elements with nonlinear material properties are used to construct ligaments (Figure 2A), including the anterior longitudinal ligament (ALL), posterior longitudinal ligament (PLL), ligamentum flavum (LF), interspinous ligament (ISL), supraspinous ligament (SSL), capsular ligament (CL), and intertransverse ligament (ITL). According to the literature, the material properties in Table 1 are assigned to each ligament and structure in the material library, including the cortical bone, cancellous bone, nucleus pulposus, annulus fibrosus, endplate, and articular cartilage. The contact type between the facet joint cartilage and the vertebral surface is set as “No separation”, and the contact gap range of the facet joint cartilage surface is set to 0.1 mm. According to the reference (Sun et al., 2024), the contact between the surface of the lumbar intervertebral disc and the upper and lower endplates of each vertebral body is defined as the “bonded” mode. The upper and lower endplates of each vertebral body are taken as the master surface, and the upper and lower surfaces of the intervertebral disc are taken as the slave surface. Finally, mesh the model, set the mesh division method for automatic division, set the mesh size by inserting the size control, set the edge length of the larger vertebrae and fibrous rings and other components to 2mm, and set the edge length of the smaller small articular cartilage and end plate components to 1 mm (Figure 2B).

**TABLE 1:** Material parameters for finite element modeling.

Parts	Young modulus/MPa	Poisson’s ratio	Sectional area/mm2	References
Cortical bone	12,000	0.3		Zhang et al. (2022)
Cancellous bone	100	0.2		Zhang et al. (2022)
Cartilage endplate	1000	0.4		Bereczki et al. (2021)
Annulus fiber	4.2	0.45		Zhang et al. (2021)
Nucleus pulposus	1	0.499		Zhang et al. (2021)
Articular facet joints	10	0.4		Wu et al. (2023)
ALL	20	0.3	60	Huang et al. (2023)
PLL	20	0.3	21	
LF	19.5	0.3	40	
SSL	15	0.3	30	
ISL	12	0.3	40	
ITL	50	0.3	10	
CL	7.5	0.3	67.5	

### 2.4 Boundary conditions and load settings

**FIGURE 3:**
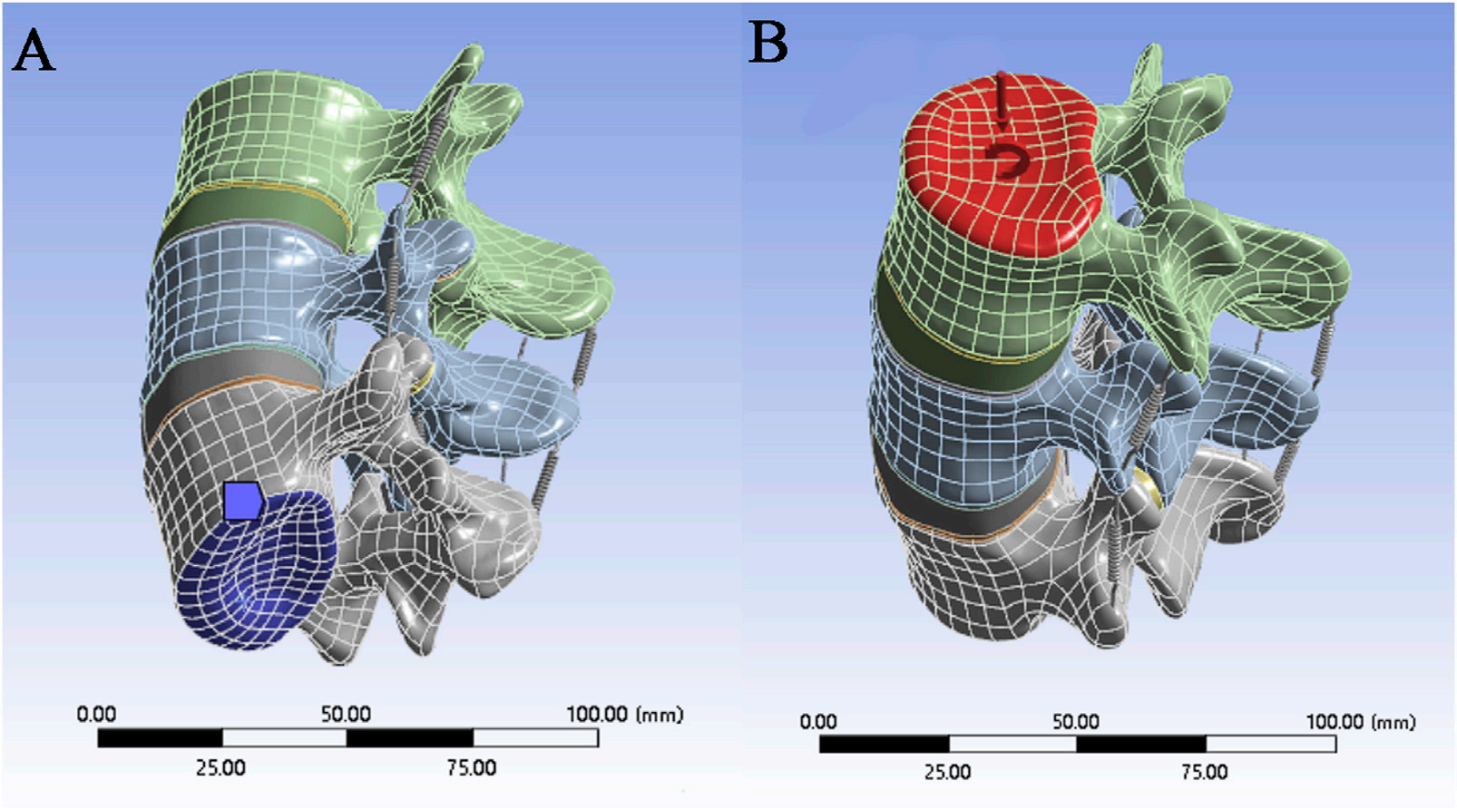
**(A)** Securing the bottom of the model; **(B)** Applying forces and moments to the top of the model.

When the model is in a neutral position, the bottom of L5 is subject to fixed constraints, whereas the upper surface of the L3 vertebral body, where pressure and torque are applied, is unconstrained (Figure 3). When the magnitude and direction of the load are set, a “component” is selected to set the load,the stress caused by the human body weight on the lumbar spine under different conditions is simulated, and the load is evenly transferred to the nodes on the surface. Pressure and torque are applied to the upper surface of the L3 vertebral body. The pressures and torques were 1175 N and 7.5 N·m in flexion; 500 N and 7.5 N·m in extension; 700 N and 7.8 N·m in lateral flexion; and 720 N and 5.5 N·m in rotation, where the pressures and torques represent the body weight and dynamic forces (Rohlmann et al., 2009; Dreischarf et al., 2011; Dreischarf et al., 2012).

### 2.5 Observation indicators

The movement of the lumbar spine was simulated under six working conditions: forward flexion, backward extension, left and right lateral bending, and left and right rotation. The changes in the von Mises stress of L3, L4, and L5 in the seven models were observed, the changes in the von Mises stress of the L4–5 and L4–5 intervertebral discs were measured, and the changes in the von Mises stress of the articular cartilage between the facet joints on both sides of L4–5 were observed.

## 3 Results

### 3.1 Model validation

**FIGURE 4:**
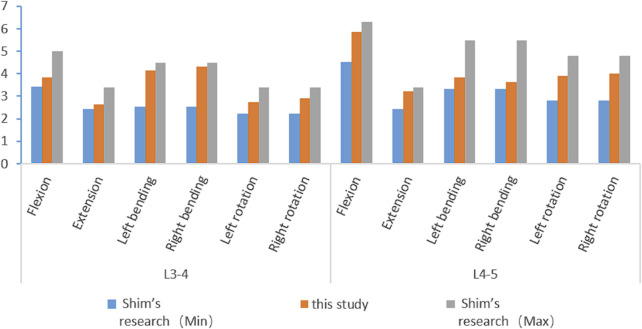
Comparison with Shim’s experimental data.

Different loading directions, such as flexion, extension, left/right bending, and left/right rotation, are applied to the vertebral body model to obtain the range of motion data of the L3--L5 segment. The results are compared with the biomechanical experimental data of previous studies (Shim et al., 2008) and show good consistency (Figure 4). This confirms the accuracy and reliability of the model and proves its suitability for subsequent simulation studies.

### 3.2 Von mises stress changes in the L4 vertebral body

**FIGURE 5:**
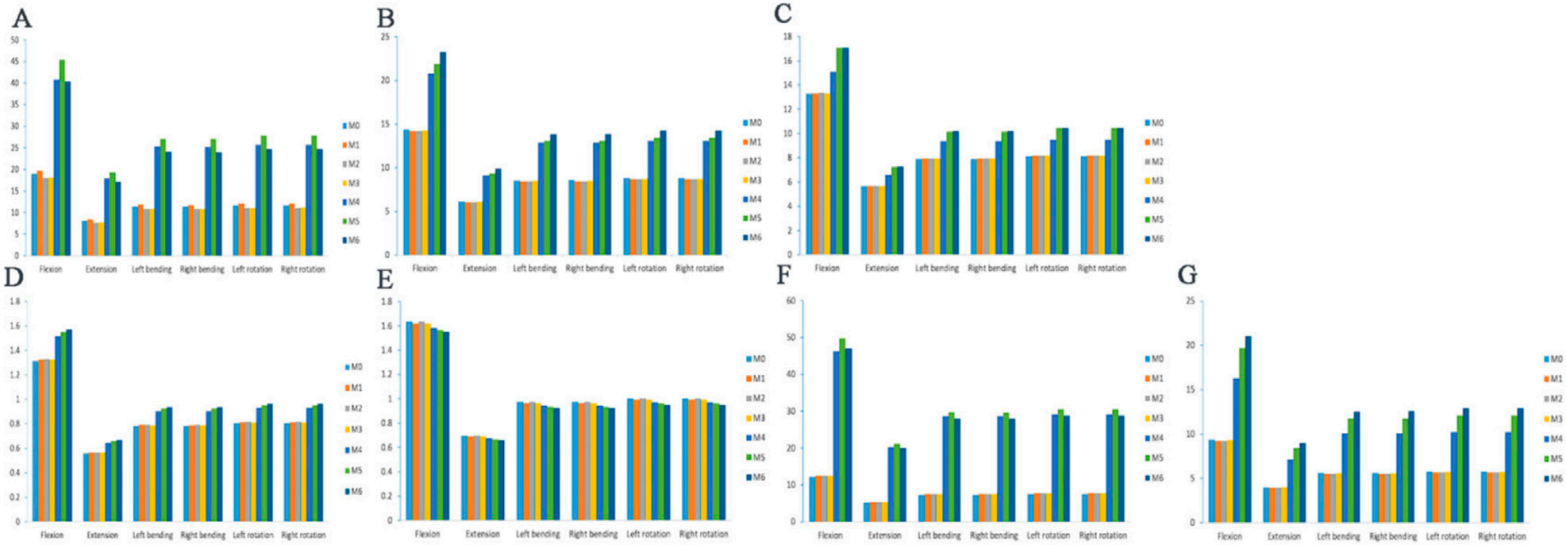
Maximum von Mises stress of the vertebral body, annulus fibers and articular cartilage. **(A)** L4 vertebral body stress; **(B)** L3 vertebral body stress; **(C)** L5 vertebral body stress; **(D)** L3-4 intervertebral disc stress; **(E)** L4-5 intervertebral disc stress; **(F)** L3-4 left articular process stress; **(G)** L3-4 right articular process stress.

In the six working conditions, compared with M0, L4 vertebral stress was elevated in the M1 model, L4 vertebral stress was reduced in the M2 and M3 models, but none of them was statistically significant (*P* > 0.05), and L4 vertebral stress was significantly elevated in the M4, M5, and M6 models (*P* < 0.05). The maximumstresses of the L4 vertebral body under the six load conditions are 45.334, 19.319, 27.038, 27, 27.786 and 27.796 MPa, respectively (Figure 5A).

### 3.3 Von mises stress changes in the L3 and L5 vertebral bodies

The stress distributions of the L3 vertebrae of the seven models at the six working conditions were as follows: at the six working conditions, the difference in the L3 vertebrae stress was not statistically significant in the M1, M2, and M3 models compared with M0 (*P* > 0.05), and the L3 vertebrae stress was significantly higher in the M4, M5, and M6 models (*P* < 0.05). The stress distributions of the L5 vertebrae of the seven models at the six working conditions were as follows: at the six working conditions, the difference in the stress of the L5 vertebrae was not statistically significant (*P* > 0.05) in the models M1, M2, and M3 compared to M0, and the stress of the L5 vertebrae was significantly higher (*P* < 0.05) in the models M4, M5, and M6, but the difference in the stress of the L5 vertebrae was not statistically significant (*P* > 0.05) in the models M5 and M6. Under six load conditions, the maximum stresses of the L3 vertebral body are 23.232, 9.893, 13.841,13.846, 14.239 and 14.239 MPa, respectively (Figure 5B), and the maximum stresses of the L5 vertebral body are 17.097, 7.277, 10.186, 10.187, 10.477 and 10.478 MPa, respectively (Figure 5C).

### 3.4 Von mises stress changes in the intervertebral disc

The stress distributions of the L3-4 intervertebral discs of the seven models in the six working conditions were as follows: in the six working conditions, the difference of L3-4 intervertebral disc stress was not statistically significant in the M1, M2, and M3 models compared with that in the M0 (*P* > 0.05), and the stress of the L3-4 intervertebral discs in the M4, M5, and M6 models was significantly elevated (*P* < 0.05), but there was no significant difference among the three groups of models (*P* > 0.05). Under six load conditions, the maximum stresses of the L3-4 intervertebral disc are 1.571, 0.669, 0.936, 0.935, 0.963 and 0.963 MPa, respectively (Figure 5D).

The stress distributions of the L4-5 intervertebral discs of the seven models under the six working conditions were as follows: under the six working conditions, the difference in the L4-5 intervertebral disc stresses in the M1, M2, and M3 models was not statistically significant compared with that in the M0 model (*P* > 0.05), and the stresses of the L4-5 intervertebral discs in the M4, M5, and M6 models were gradually decreasing, but not statistically significant (*P* > 0.05). Under six load conditions, the minimum stresses of the L4-5 intervertebral disc are 1.549, 0.659, 0.924, 0.923, 0.949 and 0.950 MPa, respectively (Figure 5E).

### 3.5 Von mises stress changes in L3–L4 articular cartilage

In the six working conditions, compared with M0, the difference of L3-4 left synovial joint stress was not statistically significant (*P* > 0.05) in M1, M2, and M3 models, and the L3-4 left synovial joint stress was significantly higher (*P* < 0.05) in M4, M5, and M6 models, but there was no significant difference between M4 and M6 models (*P* > 0.05). In the six working conditions, compared with M0, the difference of L3-4 right synaptic joint stress in M1, M2 and M3 models was not statistically significant (*P* > 0.05), and the L3-4 left synaptic joint stress in M4, M5 and M6 models was gradually increased (*P* < 0.05),and there was a statistically significant difference between the three groups of models (*P* < 0.05). Under six load conditions, the maximum stresses of the left facet joint of L3-4 are 49.739, 21.2, 29.665, 29.627, 30.494 and 30.492 MPa, respectively (Figure 5F), and the maximum stresses of the right facet joint are 21.021, 8.967, 12.522, 12.544, 12.887 and 12.894MPa, respectively (Figure 5G). When the two sides are compared, the stress of the articular cartilage on the right side of each model is significantly lower than that on the left side, and the stress change shows an asymmetric trend.

## 4 Discussion

An analysis of the seven models revealed that when the degree of resection of the superior facet joint during foraminaloplasty exceeds 1/2, the stresses on the L3, L4 and L5 vertebral bodies under the six working conditions all increase significantly, and the greater the resection volume is, the greater the stress. The increase in stress is the greatest during forward flexion, and the increase in stress is the smallest during backward extension. The increased stress in the vertebral body during left/right lateral flexion and left/right rotation is essentially the same. This finding indicates that extensive resection of the facet joint may increase the degree of degeneration of the responsible segment and adjacent segment vertebral bodies. Shi et al. (Shi et al., 2021) reported that when a 15 mm endoscope is used to resect the superior facet joint, the surgical segment increases significantly during backward extension, right flexion, and left and right rotations. The adjacent segment only increases slightly during backward extension. After resection with a 7.5 mm or 10 mm endoscope, the pressure applied to the adjacent segment vertebral body was similar to that applied to the unresected vertebral body. Therefore, the greater the resection volume of the superior facet joint is, the greater the impact on the adjacent segment vertebral body.

Wu et al. (Wu et al., 2023) reported significant stress differences in the intervertebral discs of surgical segments, whereas no obvious stress changes were observed in the intervertebral discs of adjacent segments. A study (Xie et al., 2020) showed that when the L5 superior articular process shaped model was in left and right rotation, the biggest stress of the L4/5 disc increased slightly. However, no matter which way the L5 superior articular process or the L4 inferior articular process of model was shaped, the stress impact of the L3/4 disc was small. However, no matter which way the L5 superior articular process or the L4 inferior articular process of model was shaped, the stress impact of the L3/4 disc was small. This study revealed that when the degree of resection of the superior facet joint exceeds 1/2, the stress on the L3-4 intervertebral disc in the seven models increases. However, in the L4-5 intervertebral disc, as the resection volume of the facet joint increases, the stress of the intervertebral disc gradually decreases. This finding indicates that extensive resection of the facet joint may increase degeneration of the intervertebral disc in the responsible segment but has no obvious effect on the intervertebral disc in the adjacent segment. Shi et al. (Deyo and Weinstein, 2001) reported that when S1 superior facet joint foraminoplasty is greater than 3/5, the stress of the intervertebral disc in the surgical segment significantly increases in most movement directions, which is similar to our research results.

When the degree of resection of the articular process of the superior articular joint exceeds 1/2, the stress on the articular cartilage on both sides increases significantly. Moreover, the stress increase amplitude of the articular cartilage on the left side was greater than that on the right side. The stress on both sides shows an asymmetric change. The greater the forming amplitude is, the greater the stress on the contralateral articular cartilage. This may lead to long-term effects such as degenerative scoliosis. This suggests that when performing facet joint foraminoplasty, it is best to be close to the medial side and reduce the grinding of the facet joint as much as possible on the premise of sufficient decompression. Wu et al. (Wu et al., 2023) reported that after L4/5 foraminal plasty, the stresses of the L3/4 and L5/S1 facet joints decreased, whereas the stresses of the L4/5 facet joints generally tended to increase. Significant asymmetric stress changes in the bilateral facet joints were observed in all three segments, especially during bilateral rotational movements, suggesting that unnecessary and excessive resection should be avoided in PTED to reduce the incidence of low back pain and the risk of postoperative degeneration. Some studies have shown that compared with resection alone, simultaneous resection of the tip and base of the superior articular process increases the stress on the facet joints in various motion states (Wu et al., 2024). In this study, we found that when the articular cartilage is not damaged, the stress of the facet joint does not significantly change and has little impact on spinal stability. This finding indicates that when performing foraminal plasty, damage to the articular cartilage should be avoided as much as possible.

L3-4 segmental foraminoplasty is a key technique in spine surgery for treating nerve compression in lumbar spine. By enlarging the narrowed intervertebral foramen, the mechanical compression of L3-4 nerve roots can be relieved, effectively relieving the symptoms of lumbar and leg pains and numbness,and it is especially suitable for nerve compression caused by herniated discs and osteophytes, etc. Compared with traditional open surgery, this technique can be accomplished through minimally invasive approaches such as foraminoscopy. Compared with traditional open surgery, this technique can be accomplished through minimally invasive approaches such as intervertebral foramenoscopy, which is characterized by small incisions, low bleeding, and fast postoperative recovery, which can reduce the damage to spinal stability and lower the risk of complications such as infection and scar adhesion. With the continuous development of minimally invasive techniques, vertebroplasty also provides important support for the stepwise treatment of lumbar degenerative diseases.

This study also has the following shortcomings. First, the finite element model established in this study simplifies the muscles connected to the lumbar spine and the weight of the upper body, and there is still a certain difference from the real human body. Second, factors associated with the annulus fibrosus, such as annulus fibrosus damage, reduced nucleus pulposus volume, and endplate damage caused by intervertebral disc resection, are not considered. Moreover, the formation of scar tissue in the surgical area and the ingrowth of new bone tissue after surgery also have a certain impact on the stability of the lumbar spine. Third, the finite element model analysis in this study is a one-time load study and cannot systematically analyze the impact of fatigue loading on the biomechanics of the lumbar spine. Although the spinal finite element model is increasingly in line with human anatomy compared with previous designs, more refinements are still needed to obtain more accurate shapes and physical properties to increase the biomechanical application range of personalized finite element spinal models.

## 5 Conclusion

In this study, a normal L3‒L5 segment finite element model was established; on this basis, a facet joint tip foraminoplasty model, a 1/3 facet joint foraminoplasty model, a 1/2 facet joint foraminoplasty model, a 2/3 facet joint foraminoplasty model, a 3/4 facet joint foraminoplasty model and a complete resection model of the superior facet joint were established. The model is successfully verified, the prediction results are credible, and the model can be used for biomechanical analysis and surgical condition simulation. When facet joint foraminoplasty under the combined action of large-channel endoscopy and visualized trephine exceeds 1/2, it increases the stress of the vertebral body, intervertebral disc and facet joint of the surgical segment, causes asymmetric stress on the facet joints on both sides, and may cause iatrogenic instability; however, it does not cause degeneration of the intervertebral disc in adjacent segments. Therefore, when performing foraminoplasty at the L3-4 segment, the superior articular eminence should not be removed more than1/2 to avoid lumbar instability.

## Data Availability

The original contributions presented in the study are included in the article/supplementary material, further inquiries can be directed to the corresponding author.
